# Induction of Liver Steatosis in BAP31-Deficient Mice Burdened with Tunicamycin-Induced Endoplasmic Reticulum Stress

**DOI:** 10.3390/ijms19082291

**Published:** 2018-08-04

**Authors:** Zhenhua Wu, Fan Yang, Shan Jiang, Xiaoyu Sun, Jialin Xu

**Affiliations:** Institute of Biochemistry and Molecular Biology, College of Life and Health Sciences, Northeastern University, Shenyang 110169, China; wuzhenhuauwm@163.com (Z.W.); yangfan118920@163.com (F.Y.); jiangshan33556@163.com (S.J.); sxy18504189131@163.com (X.S.)

**Keywords:** BAP31, Tunicamycin, liver steatosis, ER stress, VLDL secretion

## Abstract

Endoplasmic reticulum (ER) stress is highly associated with liver steatosis. B-cell receptor-associated protein 31 (BAP31) has been reported to be involved in ER homeostasis, and plays key roles in hepatic lipid metabolism in high-fat diet-induced obese mice. However, whether BAP31 modulates hepatic lipid metabolism via regulating ER stress is still uncertain. In this study, wild-type and liver-specific BAP31-depleted mice were administrated with ER stress activator of Tunicamycin, the markers of ER stress, liver steatosis, and the underlying molecular mechanisms were determined. BAP31 deficiency increased Tunicamycin-induced hepatic lipid accumulation, aggravated liver dysfunction, and increased the mRNA levels of ER stress markers, including glucose-regulated protein 78 (*GRP78*), X-box binding protein 1 (*XBP1*), inositol-requiring protein-1α (*IRE1α*) and C/EBP homologous protein (*CHOP*), thus promoting ER stress in vivo and in vitro. Hepatic lipid export via very low-density lipoprotein (VLDL) secretion was impaired in BAP31-depleted mice, accompanied by reduced Apolipoprotein B (*APOB*) and microsomal triglyceride transfer protein (*MTTP*) expression. Exogenous lipid clearance was also inhibited, along with impaired gene expression related to fatty acid transportation and fatty acid β-oxidation. Finally, BAP31 deficiency increased Tunicamycin-induced hepatic inflammatory response. These results demonstrate that BAP31 deficiency increased Tunicamycin-induced ER stress, impaired VLDL secretion and exogenous lipid clearance, and reduced fatty acid β-oxidation, which eventually resulted in liver steatosis.

## 1. Introduction

Nonalcoholic fatty liver disease (NAFLD) refers to a wide spectrum of hepatic pathology, ranging from a simple liver steatosis (>5% of an intrahepatic accumulation of fat) to steatohepatitis [1]. NAFLD is the most common liver disorder in Western industrialized countries, with an estimated prevalence rate up to 25% worldwide and 35% in the United States [2,3]. The economic burden of NAFLD is likely to increase in the United States and Europe. Over 64 million people are projected to have NAFLD in the United States, with annual medical costs of about $103 billion ($1613 per patient). Also, about 52 million people in Germany, France, Italy and the United Kingdom are supposed to have NAFLD, with annual cost of €35 billion (from €354 to €1.163 per patient) [4]. Endoplasmic reticulum (ER) stress has been implicated in the development of steatosis and the later stages of steatohepatitis and hepatocarcinoma [5]. Attenuating ER stress provides opportunities for pharmacological intervention for NAFLD [6]. Thus, there is an urgent need to understand the pathogenesis of liver steatosis regarding ER stress modulation and to identify new therapeutic molecular targets.

Stimuli disrupt ER homeostasis and result in unfolded protein accumulation, which induces unfolded protein response (UPR) activation. Disruption of ER protein processing is sensed by three conserved transmembrane proteins: protein kinase RNA-like endoplasmic reticulum kinase (PERK), inositol-requiring protein-1α (IRE1α), and activating transcription factor (ATF) 6. ER stress triggers a cascade reaction of transcriptional and translational events that restore ER homeostasis, promoting cell survival and adaptation. However, prolonged ER stress causes the physiological mechanism to produce pathological consequences, including insulin resistance, hepatic lipid accumulation, inflammation and apoptosis. Depletion of ATF6α promotes ER stress response, aggravated liver dysfunction, reduced fatty acid oxidation and very low-density lipoprotein (VLDL) formation, resulting in hepatic lipid accumulation and liver steatosis in mice [7]. IRE1α represses the expression of CCAAT/enhancer-binding protein (C/EBP) β, C/EBPδ, peroxisome proliferator-activated receptor γ (PPARγ), and the enzymes related to triglyceride biosynthesis. Mice with hepatocyte-specific IRE1α deletion exhibit modest hepatosteatosis [8], suggesting that the three UPR branches may act in concert to prevent the development of liver steatosis. Despite recent progress, it is still uncertain how ER stress modulates hepatic lipid metabolism and what constitutes its detailed molecular mechanism. Tunicamycin (Tm) is a naturally occurring antibiotic that induces ER stress in cells by preventing the first step in the protein biosynthesis of N-linked glycans, resulting in the accumulation of misfolded proteins [9]. Tm induces ER stress, increases hepatic lipogenesis [10], and decreases VLDL assembly and secretion [11], which is why it has been widely used in the research of fatty liver disease due to the disturbance of ER homeostasis.

B-cell receptor-associated protein 31 (BAP31) is a conserved and ubiquitously expressed protein integrated into the ER membrane [12,13], and has been implicated in ER sorting of diverse membrane proteins. In addition, BAP31 has been reported to promote the vesicular transport of major histocompatibility complex (MHC) class I molecules [14], cellubrevin [13] and tetraspanins [15], suggesting that BAP31 has an important role in ER export, retention and degradation. Sustained ER stress leads to apoptosis. Cell Death Involved p53-target 1 (CDIP1)-BAP31 complex transduces apoptotic signals from ER to mitochondria, contributes to caspase-8 activation and BCL2 associated X (BAX) oligomerization, and demonstrates a mechanism for ER-mitochondrial crosstalk for ER stress-mediated apoptotic signaling [16]. Our published study reported that liver-specific deficiency of BAP31 increased sterol regulatory element-binding protein 1c (SREBP1C) activation and lipogenesis, increased lipid accumulation and hepatic inflammation, which induced insulin resistance in high-fat-diet (HFD)-induced animal model [17]. However, whether BAP31 mutation in hepatocytes induced ER stress, and then increased lipid accumulation in the liver is not completely understood. In the current study, wild-type (WT) and BAP31 conditional knockout (KO) mice were treated with Tm to induce ER stress, which increased hepatic lipid accumulation, markers of ER stress, liver steatosis, and the underlying mechanisms will be evaluated, to explore whether BAP31 plays a role in lipid accumulation, and if it prevents the development of fatty liver disease via modulating ER stress status.

## 2. Results

### 2.1. BAP31 Deficiency Promoted Liver Dysfunction after Tunicamycin Injection

**Figure 1:**
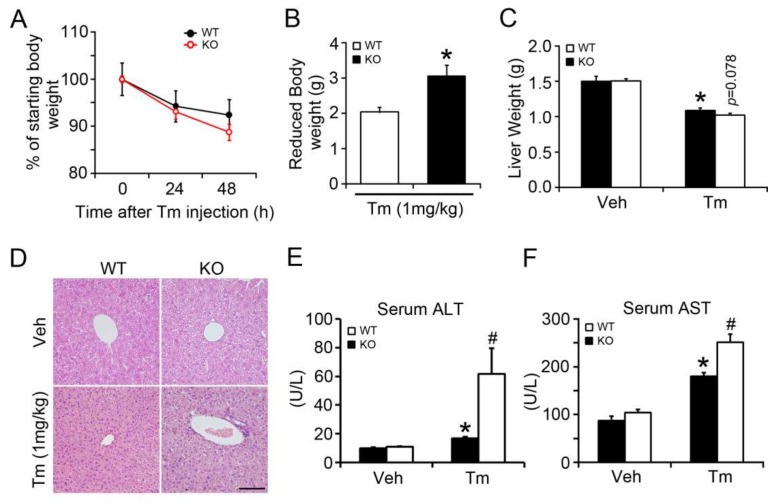
BAP31 deficiency promoted liver dysfunction after Tunicamycin injection. (**A**) Wild-type (WT) and liver-specific BAP31 knockout mice (KO) (16-week-old) were injected with Tunicamycin intraperitoneally. Body weight was monitored for 48 h and presented after normalization to that at 0 h after Tm injection. *n* = 7–8 per group. (**B**) Reduced body weight and (**C**) liver weight were monitored for each group. *n* = 7–8 per group. (**D**) Representative hematoxylin and eosin (H/E) staining of livers from WT and KO mice post Tunicamycin administration for 48 h (200×, scale bar = 100 µm). *n* = 4 per group. (**E**) Serum alanine transaminase (ALT) and (**F**) aspartate transaminase (AST) were determined in WT and KO mice injected with vehicle (Veh) or Tunicamycin (Tm) for 48 h. *n* = 7–8 per group. Data represent as mean ± SEM. *, *p* < 0.05, Tm compared with Veh group. #, *p* < 0.05, KO compared with WT mice injected with Tm.

We injected Tm intraperitoneally into WT and KO at a dose of 1 mg/kg body weight, which was previously used to analyze the effects of melatonin on ER stress-induced liver steatosis [18]. The body weight of WT and KO mice dropped dramatically after Tm administration. Further, it dropped more significantly in KO mice (Figure 1A), which exhibited 49.6% more reduction than that of WT controls (Figure 1B). Liver weights were similar between these two types of mice injected with vehicle, and were reduced in KO compared with WT mice after Tm injection (*p* = 0.078) (Figure 1C). Hematoxylin and eosin (H/E) staining revealed no significant difference initially between these two types of mice at normal conditions, although differences became apparent 48 h after Tm injection (Figure 1D). Serum alanine transaminase (ALT) and aspartate transaminase (AST), which can be used as an index of liver injury, were increased by 69% and 107% in WT mice after Tm administration. BAP31 deficiency increased the levels of ALT and AST by 266% and 39% more than that of WT mice, respectively (Figure 1E,F). These results indicate that Tm-induced liver dysfunction is specific to BAP31 conditional knockout mice.

### 2.2. BAP31 Deficiency Promoted Tunicamycin-Induced Hepatic Lipid Accumulation

**Figure 2:**
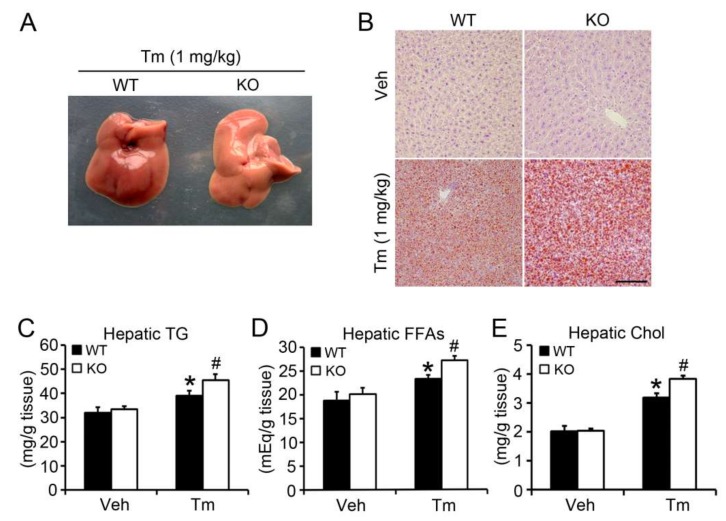
BAP31 deficiency promoted Tunicamycin-induced hepatic lipid accumulation. (**A**) Representative photo of livers from WT and KO mice post Tunicamycin administration for 48 h. (**B**) Representative Oil red O staining of livers from WT and KO mice post Tunicamycin administration for 48 h (200×, scale bar = 100 µm). *n* = 4 per group. Hepatic (**C**) triglycerides (TG), (**D**) free fatty acids (FFAs), and (**E**) cholesterol (Chol) were quantified spectrophotometrically. *n* = 7–8 per group. Data represent as mean ± SEM. *, *p* < 0.05, Tm compared with Veh group. #, *p* < 0.05, KO compared with WT mice injected with Tm.

The livers from KO mice were grayer than those of WT mice, indicating enhanced lipid accumulation (Figure 2A). Oil Red O staining showed no obvious difference between WT and KO mice injected with vehicle. Tm administration increased the red staining, and was higher in KO than WT mice, suggesting that BAP31 deficiency increased Tm-induced liver steatosis (Figure 2B). To confirm BAP31’s function in lipid metabolism in the liver, lipid particles were purified and quantified spectrophotometrically. There was no obvious difference of hepatic triglycerides (TG) between WT and KO mice injected with vehicle. Tm increased hepatic TG significantly. BAP31 deficiency accelerated the increase, which was 17% higher than that of WT mice (Figure 2C). Similar changes in hepatic free fatty acids (FFAs) and cholesterol (Chol) were observed. Tm administration increased the FFAs and Chol content, and BAP31 deficiency led to an even greater increase, with FFAs and Chol 17% and 21% higher than in WT mice, respectively (Figure 2D,E). Next, the profiles of serum metabolites were determined. Tm administration reduced serum glucose, TG, FFAs, Chol, high-density lipoprotein cholesterol (HDL-C) and low-density lipoprotein cholesterol (LDL-C) significantly. No difference of glucose, TG, Chol, HDL-C and LDL-C was observed between WT and KO mice after Tm administration. Only FFAs were increased in KO when compared with WT mice (Table 1).

### 2.3. BAP31 Deficiency Reduced Lipogenic Gene Expression in Tunicamycin-Injected Mice

**Figure 3:**
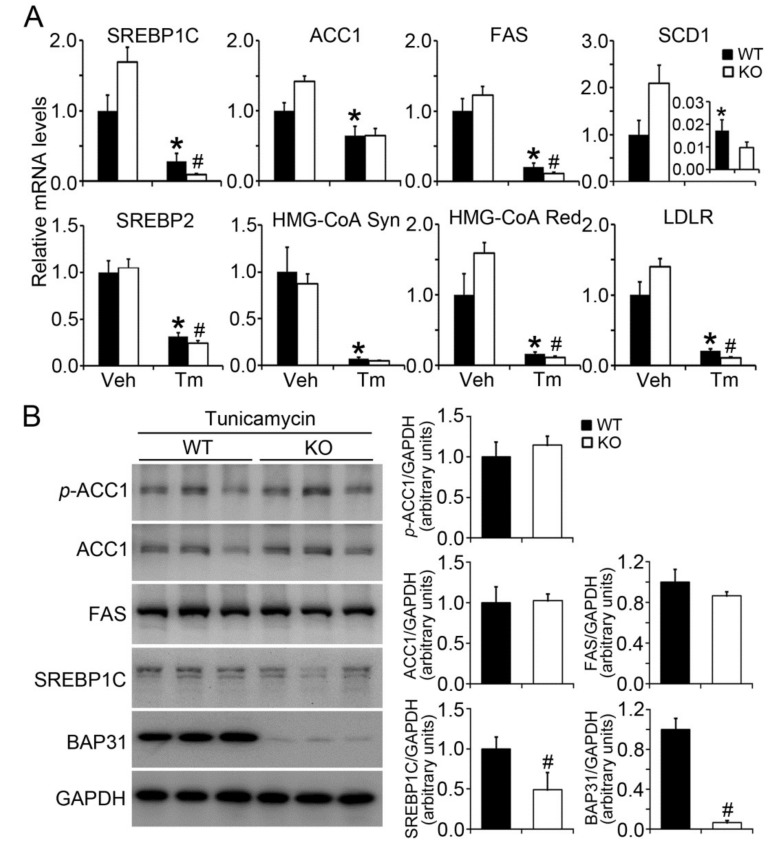
BAP31 deficiency reduced lipogenic gene expression in Tunicamycin-injected mice. (**A**) Total RNA was extracted from the liver tissues. The relative mRNA levels were determined by quantitative real-time PCR and normalized with *18S* rRNA levels. *n* = 7–8 per group. (**B**) Immunoblot analysis of BAP31, SREBP1C, FAS, ACC-1, *p*-ACC1 in livers from mice injected with Tunicamycin for 48 h. Data represent as mean ± SEM. *, *p* < 0.05, Tm compared with Veh group. #, *p* < 0.05, KO compared with WT mice injected with Tm.

Sterol regulatory element-binding protein (SREBP) 1C is well recognized as a key transcription factor for the regulation of lipogenesis [19]. It was reduced in WT mice after Tm administration, and was reduced more in KO mice. The target genes of acetyl-CoA carboxylase 1 (*ACC1*), fatty acid synthase (*FAS*), and stearoyl-CoA desaturase-1 (*SCD1*) were significantly decreased after Tm administration. Compared with WT controls, *FAS* deceased significantly, but *SCD1* decreased insignificantly in KO mice. Additionally, *SREBP2* was reduced after Tm administration, especially in KO mice. Similar changes were found for 3-Hydroxy-3-Methylglutaryl-CoA synthase (*HMG-CoA Syn*), 3-hydroxy-3-methyl-glutaryl-coenzyme A reductase (*HMG-CoA Red*), and low-density lipoprotein receptor (*LDLR*) genes, which were decreased after Tm administration. Further, the mRNA levels decreased more in KO than in WT mice (Figure 3A). BAP31 protein levels were significantly reduced in KO mice, exhibiting only 6% as that of WT controls. SREBP1C protein levels were decreased by 51% in KO mice. There was no obvious difference for ACC1 protein levels between these two types of mice, with a slight increase of the phosphorylation levels of ACC1 (*p*-ACC1) and a slight decrease of FAS protein levels in KO mice, demonstrating that BAP31 depletion reduced lipogenesis in mice (Figure 3B).

### 2.4. BAP31 Deficiency Impaired VLDL Secretion and Exogenous Lipid Clearance, Reduced the Gene Expression Related to Fatty Acid β-Oxidation

**Figure 4:**
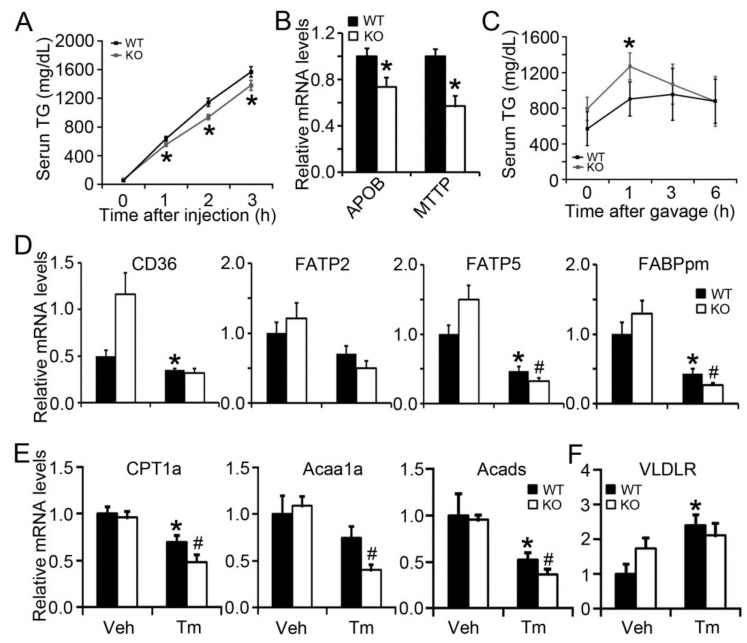
BAP31 deficiency impaired VLDL secretion and exogenous lipid clearance, reduced the gene expression related to fatty acid β-oxidation. (**A**) BAP31 deficiency impaired VLDL secretion. (**B**) BAP31 deficiency decreased *APOB* and *MTTP* mRNA levels in mice livers. *n* = 7 to 8 per group. (**C**) BAP31 deficiency reduced exogenous lipid clearance in mice. *n* = 6 to 7 per group. *, *p* < 0.05, KO mice compared with WT mice. The relative mRNA levels of (**D**) *CD36*, *FATP2*, *FATP5*, *FABPpm*, (**E**) *CPT1a*, *Acaa1a*, *Acads*, and (**F**) *VLDLR* were determined by quantitative real-time PCR and normalized with *18S* rRNA levels. *n* = 7 to 8 per group. Data represent as mean ± SEM. *, *p* < 0.05, Tm compared with Veh group. #, *p* < 0.05, KO compared with WT mice injected with Tm.

Hepatic steatosis has been reported in patients with familial hypobetalipoproteinemia (mutation in APOB 100), due to impaired VLDL assembly and reduced lipid secretion from the liver [20]. Tyloxapol has commonly been used to inhibit lipoprotein lipase [21]. When the lipoprotein lipase is inhibited, the plasma lipid is mostly sourced from lipid export via VLDL secretion. Serum TG increased continuously in WT mice after tyloxapol administration, but was lower in KO mice at 1-, 2-, and 3-h timepoints, suggesting reduced VLDL secretion in these mice and that BAP31 deficiency impaired VLDL secretion (Figure 4A). Apolipoprotein B (*APOB*) and microsomal triglyceride transfer protein (*MTTP*), which are responsible for VLDL assembly and secretion, were significantly reduced in KO mice, confirming that BAP31 deficiency impairs VLDL secretion (Figure 4B). Exogenous lipid clearance rate was also evaluated in WT and KO mice. KO mice exhibited a higher plasma lipid content than the WT controls, suggesting that BAP31 deficiency impairs exogenous lipid clearance (Figure 4C). Fatty acid transporters, including cluster of differentiation 36 (*CD36*), fatty acid transport protein 5 (*FATP5*), and membrane-associated fatty acid binding protein (*FABPpm*), which are responsible for fatty acid transportation across the plasma membrane of hepatocytes [22], were significantly reduced after Tm administration. *FATP5* and *FABPpm* were decreased more in KO than in WT mice. Only a negligible difference for *CD36* and *FATP2* was found between WT and KO mice after Tm administration (Figure 4D). Compared with WT controls treated with Tm, the mRNA levels of genes related to fatty acid β-oxidation, including carnitine palmitoyltransferase 1a (*CPT1a*), 3-ketoacyl-CoA thiolase (*Acaa1a*), and acyl-CoA dehydrogenases (*Acads*), were significantly reduced, suggesting that the process of fatty acid β-oxidation was inhibited in BAP31 KO mice (Figure 4E). The mRNA levels of very low-density lipoprotein receptor (*VLDLR*) was induced due to Tm administration, but exhibits no difference between WT and KO mice under ER stress (Figure 4F).

### 2.5. BAP31 Deficiency Increased Tunicamycin-Induced ER Stress

**Figure 5:**
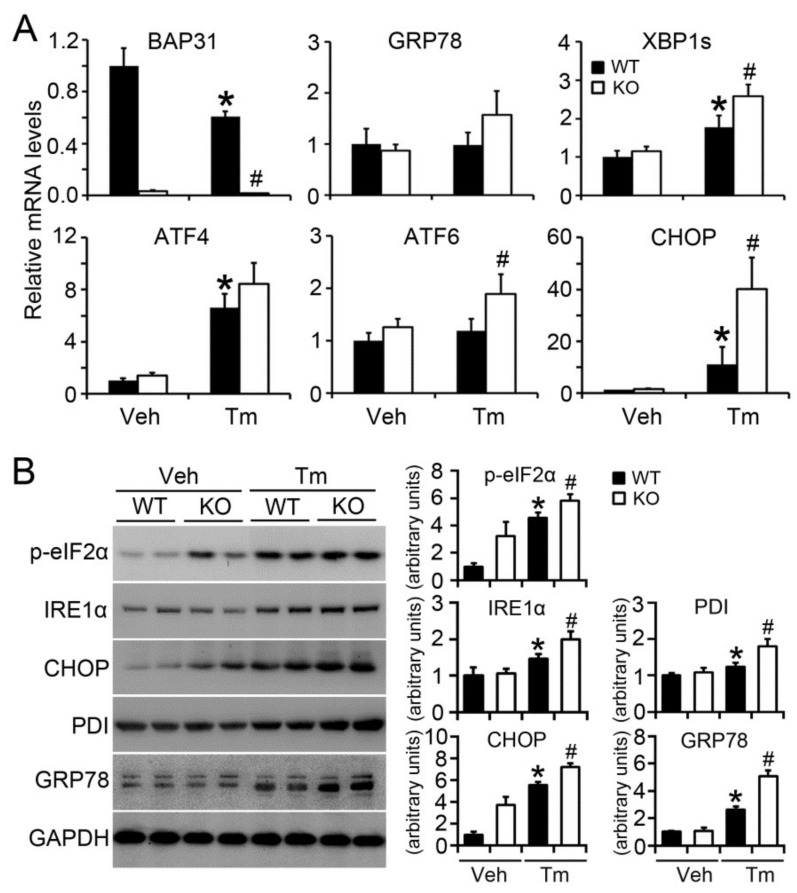
BAP31 deficiency increased Tunicamycin-induced ER stress. (**A**) The relative mRNA levels of ER stress markers, including *GRP78*, *XBP1s*, *ATF4*, *ATF6* and *CHOP*, was determined in mice livers injected with vehicle (Veh) of Tunicamycin (Tm) for 48 h. *n* = 7 to 8 per group. (**B**) Immunoblot assay of *p*-eIF2α, IRE1α, CHOP, PDI and GRP78 in mice livers injected with vehicle (Veh) or Tunicamycin (Tm) for 48 h. GAPDH was used as a loading control. Data represent as mean ± SEM. *, *p* < 0.05, Tm compared with Veh group. #, *p* < 0.05, KO compared with WT mice injected with Tm.

BAP31 mRNA levels were reduced due to Tm administration. *XBP1s* (spliced *XBP1*) and C/EBP homologous protein (*CHOP*) mRNA levels were significantly increased, along with insignificant increase of glucose-regulated protein 78 (*GRP78*) and *ATF4*, suggesting enhanced Tm-induced ER stress in KO mice (Figure 5A). Tm increased the protein levels of ER stress markers, including IRE1α, CHOP, protein disulfide isomerase (PDI) and GRP78, and increased the phosphorylation levels of eukaryotic translation initiation factor 2α (*p*-eIF2α). BAP31 deficiency even enhanced the increase, confirming that BAP31 deficiency increased Tm-induced ER stress (Figure 5B).

### 2.6. BAP31 Deficiency Increased Inflammatory Response in Mice upon Tunicamycin Injection

**Figure 6:**
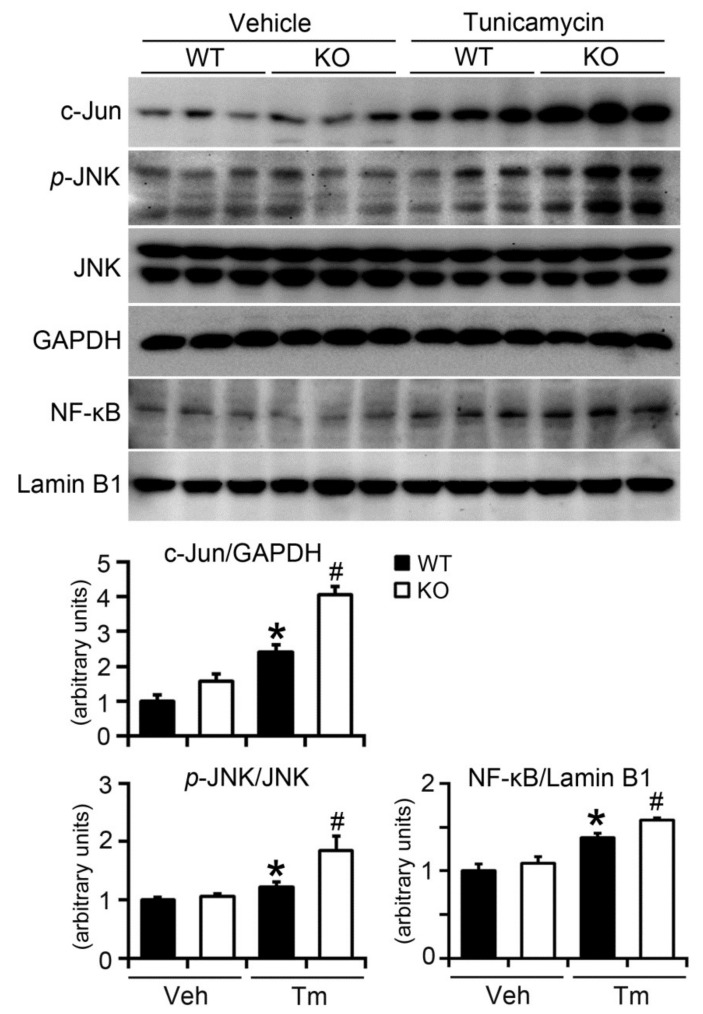
BAP31 deficiency increased inflammatory response in mice upon Tunicamycin injection. Immunoblot analysis of c-Jun, *p*-JNK, and NF-κB in mice livers. GAPDH and Lamin B1 were used as a loading control. Data represent as mean ± SEM. *, *p* < 0.05, KO compared with WT mice injected with Tm. #, *p* < 0.05, KO compared with WT mice injected with Tm.

Increased ER stress not only regulates lipid metabolism in the liver, but also interacts with inflammatory cascades at various stages, including activation of C-Jun N-terminal kinases (JNK) and nuclear factor kappa-light-chain-enhancer of activated B cells (NF-κB) signaling pathways [23]. BAP31 deficiency increased c-Jun protein levels significantly in vehicle-treated mice. Tm increased c-Jun and *p*-JNK protein levels and the increase was even higher in KO mice, suggesting that there is a greater inflammatory response in KO mice livers after Tm administration. Thus, we next purified the nuclear fractions from the livers and performed Western blot against the transcription factor of NF-κB. There was a slight increase of nuclear NF-κB content in KO compared with that of WT vehicle-treated mice. Tm promoted nuclear localization of NF-κB. BAP31 deficiency even enhanced the nuclear localization, suggesting that NF-κB signaling was activated, and thus increased the inflammatory response in mice (Figure 6).

### 2.7. BAP31 Deficiency Induced ER Stress In Vitro

**Figure 7:**
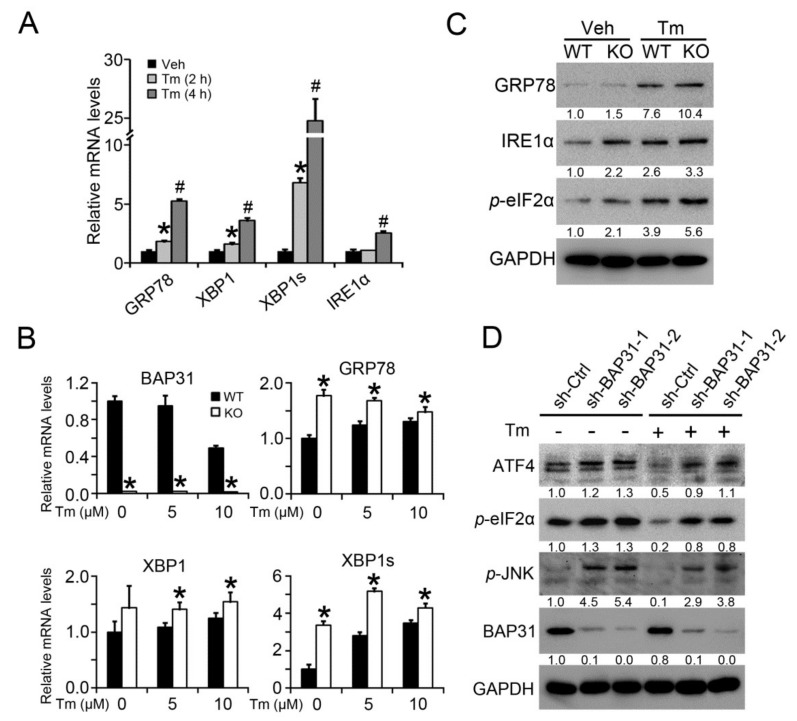
BAP31 deficiency induced ER stress in vitro. Primary hepatocytes were isolated from male WT and KO mice. (**A**) 24 h after the attachment, cells were treated with Tunicamycin (5 μM) for 2 or 4 h, the mRNA levels of *GRP78*, *XBP1*, *XBP1s* and *IRE1α* were determined. Data represent as mean ± SEM. *, *p* < 0.05, Tm (2 h) compared with Veh. #, *p* < 0.05, Tm (4 h) compared with Veh. (**B**) Primary hepatocytes were treated with Tunicamycin (5 and 10 μM) for 4 h, the mRNA levels of *BAP31*, *GRP78*, *XBP1* and *XBP1s* were determined. Data represent as mean ± SEM. *, *p* < 0.05, KO hepatocytes compared with WT. (**C**) Primary hepatocytes were treated with Tunicamycin (5 μM) for 12 h. The protein levels of GRP78, IRE1α, *p*-eIF2α were determined. (**D**) HepG2 cells with targeted disruption of BAP31 were treated with Tunicamycin (5 μM) for 12 h. The protein levels of ATF4, *p*-eIF2α, *p*-JNK and BAP31 were determined. GAPDH was used as a loading control. All experiments have been repeated three times individually. The result of immunoblot analysis has been normalized with control group.

Tm treatment increased the mRNA levels of ER stress markers of *GRP78*, *XBP1*, *XBP1s* and *IRE1α* in primary hepatocytes (Figure 7A). BAP31 deficiency enhanced the increase, describing by elevated mRNA levels of *GRP78*, *XBP1* and *XBP1s* in the primary hepatocytes isolated from KO mice than in WT controls (Figure 7B). Western blot analysis confirmed the observation. BAP31 deficiency increased the protein levels of GRP78, IRE1α and *p*-eIF2α in KO primary hepatocytes (Figure 7C). HepG2 cells with targeted disruption of BAP31 were treated with Tm, and then ER stress markers were evaluated. BAP31 protein levels were significantly reduced in sh-BAP31 cell lines, suggesting that BAP31 expression was disrupted. The protein levels of ATF4, *p*-eIF2α and *p*-JNK were increased in BAP31-knockdown cell lines with or without Tm treatment, further confirming that BAP31 deficiency induced ER stress (Figure 7D).

## 3. Discussion

In this study, we reported that BAP31 deficiency in hepatocytes increased ER stress when burdened with Tm, increased Tm-induced liver dysfunction and hepatic inflammation, reduced VLDL secretion and lipid clearance, decreased the gene expression related to fatty acid oxidation, and eventually increased hepatic lipid accumulation. This study reveals the links among BAP31, ER stress, and liver steatosis, and points to the protective roles of BAP31 in the development of fatty liver disease.

Tm administration increased the genes expression of ER stress markers. BAP31 deficiency further enhanced this increase, demonstrating that BAP31 depletion induced ER stress activation. BAP31 is associated with the newly synthesized client proteins of the Δ508 mutant of CFTR, and promotes the retrotranslocation from the ER and degradation by 26S proteasome system. The depletion of BAP31 reduced the proteasome degradation and permitted a significant fraction of the surviving protein to reach to the cell surface, thus increasing the misfolded protein accumulation [24]. Increased accumulation of misfolded protein induces UPR, resulting in a transient attenuation in the rate of protein synthesis and an upregulation of gene-encoded chaperons and other proteins participating in polypeptide folding, which disrupts ER function and leads to ER stress [25]. Upon ER stress, CDIP1 was induced and it enhanced the association with BAP31, promoting ER stress-induced apoptotic signaling, further revealing the important roles of BAP31 in mediating ER stress induction [16]. Depletion of *BAP31* gene in hepatocytes increased lipogenic gene expression, increased lipid synthesis and hepatic lipid accumulation, and was accompanied by increased gene expression of ER stress markers in HFD-induced obesity, showing that BAP31 deficiency induces ER stress [17]. Misfolded proteins of CFTR induced unesterified cholesterol accumulation in late endosomes and lipid dysfunction. VAPB inhibited the degradation of the Δ508 mutant of CFTR through interaction with the ring finger protein 5 (RNF5 or RMA1)-Derlin-BAP31-VAMP-associated Proteins (VCP) pathway, revealing that BAP31 is important in maintaining lipid homeostasis [26]. It was noted that mutations in BAP31 caused a severe X-linked phenotype with deafness, dystonia and disorganization in the Golgi apparatus, but failed to show any increase of UPR markers of DNA damage inducible transcript 3 (DDIT3), heat shock protein family A (Hsp70) member 5 (HSPA5, also known as GRP78) and XBP1 when the cells were treated with the ER stress inducer of Thapsigargin. Also, the authors failed to detect any exacerbated cell death, even though it has been reported that BAP31 has a role in cell death [27,28]. The possible reason is that there are contiguous mutations in the reported case, which means that not only is *BAP31* gene mutation involved in the study, but that the mutation of solute carrier family 6 member 8 (SLC6A8) also contributes and complicates the mechanisms, which makes the patient exhibit a different phenotype compared with the single mutation of *BAP31* gene in the current study. Future studies on the gain- or loss-of-function for *BAP31* gene are warranted in order to explore the detailed function of BAP31 in ER stress activation and related metabolic diseases.

Increased liver lipid accumulation is due to increased de novo lipogenesis, increased lipid uptake from the diet, reduced fatty acid oxidation, and impaired lipid export via VLDL secretion. SREBP1C is the predominant regulator of de novo lipogenesis and regulates the expression of the key lipogenic genes of *ACC1*, *FAS* and *SCD1*. Increased expression of SREBP1C, ACC1, FAS and SCD1 promoted hepatic lipid accumulation and induced liver steatosis [29]. Tm increased ER stress, induced UPR activation, suppressed the mRNA or protein levels of the lipogenic genes, and reduced lipogenesis in the liver [11,30], which is consistent with the current study. BAP31 deficiency increased Tm-induced liver steatosis, even with reduced lipogenic gene expression, suggesting that there are more complicated mechanisms involved. Patients with familial hypobetalipoproteinemia often develop fatty liver due to mutations in the *APOB* gene, which either abolishes or interferes with the translation of full-length APOB protein, thus impairing lipid export from the liver [31]. The postprandial suppression of VLDL-TG secretion was impaired in obese type 2 diabetic men when compared with lean healthy men, contributing to postprandial hypertriacylglycerolemia [32]. We demonstrated that VLDL secretion was reduced in BAP31 mutant mice, and was accompanied by reduced gene expression of *APOB* and *MTTP*, which may reduce lipid export from the liver, and thus increase hepatic lipid accumulation. We failed to explore the detailed molecular mechanism of BAP31 for regulating VLDL assembly. Whether or not BAP31 deficiency affects the formation of lipid droplets in the liver is still uncertain and should be considered in future research. It was noted that Tm administration increased *VLDLR* mRNA levels in the liver, which is in agreement with the previous study [30]. However, we failed to find any difference between WT and KO mice, suggesting that BAP31 may not modulate VLDLR expression directly. Whether VLDLR mediates BAP31 function on lipid metabolism in the liver is still unknown and needs to be clarified in the future.

Impaired lipid clearance also leads to liver steatosis. Mice with depleted C1q/TNF-related protein 12 exhibited impaired lipid clearance in response to acute lipid gavage, reduced hepatic TG secretion, and increased liver steatosis when challenged with HFD [33]. BAP31 deficiency reduced lipid clearance upon olive oil gavage, contributing to increased hepatic lipid accumulation. ER stress inhibited fatty acid oxidation and contributed to the development of liver steatosis [34]. Under ER stress conditions, ATF6α knockout mice exhibited hepatic steatosis due to the inhibition of fatty acid β-oxidation, and was accompanied by reduced expression of the related genes, including *PPARα*, *CPT1*, *CPT2* and *ACOX1* [7]. We determined that BAP31 deficiency decreased *CPT1a*, *Acaa1a* and *Acads* expression, which are responsible for peroxisomal and mitochondrial fatty acid β-oxidation, in KO mice under ER stress, suggesting that BAP31 deficiency may impair fatty acid oxidation in mice, and lead to liver steatosis.

ALT and AST are enzymes located in hepatocytes under normal conditions and are leaked into blood when the hepatocytes are injured. They have been used as the biomarkers of liver injury in patients with some degree of intact liver function [35]. Serum ALT and AST content were increased after Tm administration. BAP31 deficiency enhanced this increase, revealing increased liver dysfunction in BAP31 mutant mice, which suggests the protective role of BAP31 against Tm assault. Tm administration progressively reduced the body weight of WT and KO mice [7], and reduced even more in KO mice, demonstrating that the effects of Tm were exacerbated due to BAP31 depletion. The patients with BAP31 mutations showed an abnormal ER with a disorganized Golgi apparatus, suggesting the key roles of BAP31 in ER-to-Golgi changes, which is a function that cannot be compensated in human cells [28]. Increased ER stress is highly associated with hepatic inflammation in the pathogenesis of liver steatosis [36]. Administration of Tm increased IRE1α and PERK activation, leading to overexpression of CHOP, and then activation of hepatic inflammation by activating the NLR family pyrin domain containing 3 (NLRP3) inflammasome, which points to the links between ER stress and liver inflammation [37]. The protein levels of c-Jun, p-JNK and nuclear NK-κB were increased more in KO than in WT mice after Tm administration, thus enhancing the liver inflammatory response in KO mice. Prolonged or enhanced liver inflammation tends to initiate hepatocyte pyrotosis and apoptosis and induce liver injury in the end [38]. Elevated FFAs induced ER stress and activated the inflammatory signaling pathway in the liver [39]. In this study, we reported that the FFAs content in KO mice was significantly higher than that of WT mice when administrated with Tm, which contributed to increased ER stress and hepatic inflammatory response. The results indicate that BAP31 is important for ER homeostasis, plays a critical role in response to ER stress, and initiates a protective role against ER stress-induced liver injury.

## 4. Materials and Methods

### 4.1. Chemicals

Tunicamycin (#243578) and olive oil (#41654) were obtained from J&K Scientific (Shanghai, China). Oil Red O (#O0625) and tyloxapol (#T8761) were got from Sigma-Aldrich (St. Louis, MO, USA). Ethanol, methanol, isopropanol and other chemicals without specific illustration were obtained from Sinopharm Chemical Reagent Co., Ltd. (Shanghai, China).

### 4.2. Animals and Tunicamycin Administration

Male WT and KO (16-week-old) mice were housed under a controlled temperature (22–25 °C) with relative humidity (30–70%) and lighting (12 h, light–dark cycles) environment. Mice were injected with Tm (1 mg/kg body weight, i.p.) for 48 h. Liver tissues were collected. Blood samples were centrifuged at 5000× *g* for 30 min and then sera were collected for the next steps. All procedures were conducted in accordance with the National Institutes of Health Guidelines for the Care and Use of Laboratory Animals and were approved by the Northeastern University Animal Care and Use Committee (No. 2015(029)) in September 2015.

### 4.3. Primary Hepatocytes Isolation and Treatment

Primary hepatocytes were isolated from male WT and KO mice (3-month-old) by using a two-step collagenase perfusion method as before [40]. 1 × 10^6^ cells/well were seeded on collagen-coated 6-well plates. Four hours after attachment, cells were switched to serum-free media containing 1% ITS supplement (Invitrogen, Carlsbad, CA, USA) and cultured overnight. Hepatocytes were treated with Tm (5 and 10 μM) with the indicated time and harvested for further experiment.

### 4.4. VLDL Secretion and Exogenous Lipid Clearance Measurement

For VLDL secretion measurement, mice (8-week-old) fasted 5 h were injected with tyloxapol via tail vein (Triton WR-1339, Sigma-Aldrich, St. Louis, MO, USA) (0.5 g/kg) in 0.9% NaCl solution containing 10% WR-1339. Blood samples were collected at 0, 1, 2 and 3 h post injection and serum TG was measured [30]. For exogenous lipid clearance assay, mice (9-week-old) fasted overnight were administered olive oil via oral gavage (15 mL/kg, Sigma-Aldrich, St. Louis, MO, USA). Blood samples were collected at 0, 1, 3 and 6 h after oil administration and serum TG was measured.

### 4.5. HepG2 Cells Treatment

ShRNA lentivirus was obtained from Novobio Scientific (Shanghai, China). HepG2 cells were obtained from the Cell Resource Center, Peking Union Medical College (which is the headquarters of National Infrastructure of Cell Line Resource). HepG2 cells were infected with shRNA targeting BAP31 and the scrambled non-target negative control based on the manufacturer’s instructions. Cells were seeded to a 6-well plate for 12 h (in DMEM containing 10% FBS), and then switched to serum-free media containing 1% fatty acid-free BSA with the presence of Tm for another 12 h. Then cells were lysed with RIPA buffer and the cellular protein was purified for Western blot analysis.

### 4.6. Measurement of Serum Metabolites and Liver Extracts

Serum TG, FFAs and Chol were determined with reagent kits (Pointe Scientific, Canton, MI, USA; Wako Chemicals USA, Richmond, VA, USA). Glucose, HDL-C, LDL-C, AST and ALT were determined with reagent kits from Nanjing Jiancheng Biomedical Company (Nanjing, China). Liver tissue (50 mg) was homogenized with PBS and extracted with the mixture of chloroform–methanol (2:1; *v/v*) described as before [41]. Lipid content has been normalized with tissue weight.

### 4.7. Histopathology

Sections (5 μm) of paraffin-embedded liver were cut and stained with hematoxylin and eosin before histopathology analysis. For Oil Red O staining, frozen sections were fixed and incubated with Oil Red O solution (six parts Oil Red O stock solution and four parts H_2_O; Oil Red O stock solution is 0.5% Oil Red O in 100% isopropanol) for 15 min, then counterstained with hematoxylin and mounted in glycerin jelly (Burlington, NC, USA).

### 4.8. RNA Isolation and Real-Time PCR

Total RNA was isolated using TRIzol reagent (Thermo Fisher Scientific Inc., Waltham, MA, USA) according to the manufacturer’s instructions. Two micrograms of total RNA were converted to cDNA. The relative mRNA levels were quantified using a CFX96 Touch™ Real-Time PCR Detection System (Bio-Rad Laboratories, Hercules, CA, USA). SYBR green chemistry was used. The sequences of primers used are listed in Table S1.

### 4.9. Immunoblot Analysis

Nuclear extracts were isolated by using NE-PER kit (Pierce, Rockford, IL, USA). Liver tissue (30 mg) was homogenized with 1 mL RIPA buffer. Then centrifuged at 12,000× *g* for 15 min. The supernatants were collected. The protein concentration was measured by using BCA assay (Thermo Fisher Scientific Inc., Waltham, MA, USA). Nuclear extracts and homogenates were resolved by SDS-PAGE and then transferred to PVDF membrane. The membrane was blocked with 5% non-fat dry milk in TBST followed by incubation with primary antibodies overnight at 4 °C. Bands were visualized with Bio-Rad ChemiDoc™ Imaging Systems using an ECL detection kit. The antibodies source and dilution are listed in Table S2.

### 4.10. Statistical Analysis

Quantitative data were presented as mean ± SEM. Statistic differences were determined by a two-way ANOVA followed by a Duncan’s Multiple Range post hoc test using the SPSS 13.0 software (SPSS, Chicago, IL, USA). All statistical tests with *p* < 0.05 were considered significant.

## Figures and Tables

**Table 1:** Serum metabolites of mice after Tunicamycin administration for 48 h.

Variable	Vehicle		Tunicamycin	
	WT	KO	WT	KO
Glucose (mg/dL)	160.15 ± 9.44	176.39 ± 6.32	135.37 ± 6.05 *	123.92 ± 8.34
TG (mg/dL)	99.88 ± 11.46	91.84 ± 4.72	22.70 ± 1.63 *	22.23 ± 3.02
FFAs (mEq/L)	0.87 ± 0.07	0.76 ± 0.04	0.24 ± 0.01 *	0.31 ± 0.02 ^#^
Chol (mg/dL)	77.32 ± 3.36	88.78 ± 2.42	6.03 ± 0.78 *	6.11 ± 0.87
HDL-C (mg/dL)	53.75 ± 3.10	57.28 ± 1.80	6.17 ± 0.56 *	7.09 ± 0.75
LDL-C (mg/dL)	31.15 ± 5.90	40.30 ± 5.90	n.d.	n.d.

Serum glucose, triglycerides (TG), free fatty acids (FFAs), cholesterol (Chol), HDL-cholesterol (HDL-C), and LDL-cholesterol (LDL-C) were measured from WT and KO mice injected with vehicle or Tunicamycin for 48 h. Data are shown as mean ± SEM. *n* = 7 to 8 per group. n.d., not detectable. *, *p* < 0.05, Tm compared with Veh group. #, *p* < 0.05, KO compared with WT mice injected with Tm.

## Supplementary Materials

Supplementary materials can be found at http://www.mdpi.com/1422-0067/19/8/2291/s1. Table S1: Primer sequences for real-time PCR; Table S2: List of antibodies used in this study.

## Abbreviations

Acaa1a	3-ketoacyl-CoA thiolase
Acads	Acyl-CoA dehydrogenases
ACC1	Acetyl-CoA carboxylase 1
ATF	Activating transcription factor
ALT	Alanine transaminase
APOB	Apolipoprotein B
AST	Aspartate transaminase
BAP31	B-cell receptor-associated protein 31
Chol	Cholesterol
CD36	Cluster of differentiation 36
CHOP	C/EBP homologous protein
CPT1a	Carnitine palmitoyltransferase 1a
FABPpm	Membrane-associated fatty acid binding protein
FATP	Fatty acid transport protein
FAS	Fatty acid synthase
FFA	Free fatty acid
GRP78	Glucose-regulated protein 78
HDL-C	High-density lipoprotein cholesterol
HMG-CoA Syn	3-hydroxy-3-methylglutaryl-CoA synthase
HMG-CoA Red	3-hydroxy-3-methyl-glutaryl-Coenzyme A reductase
IRE1α	Inositol-requiring enzyme-1α
JNK	C-Jun N-terminal kinases
LDL-C	Low-density lipoprotein cholesterol
LDLR	Low-density lipoprotein receptor
MTTP	Microsomal triglyceride transfer protein
NF-κB	Nuclear factor kappa-light-chain-enhancer of activated B cells
PDI	Protein disulfide isomerase
SCD1	Stearoyl-CoA desaturase-1
SREBP	Sterol regulatory element-binding protein
Tm	Tunicamycin
TG	Triglycerides
VLDLR	Very low-density lipoprotein receptor
XBP1	X-box binding protein 1
