# Supplementary material for: Induction of Liver Steatosis in BAP31-Deficient Mice Burdened with Tunicamycin-Induced Endoplasmic Reticulum Stress
Source: Int J Mol Sci. 2018 Aug 4;19(8):2291. doi: 10.3390/ijms19082291 (PMC6121476; doi:10.3390/ijms19082291)
Supplement: Supplementary file 1 [file ijms-19-02291-s001.pdf]

Table S1. Primer sequences for real-time PCR.

Genes	Forward	Reverse
18S	AGTCCCTGCCCTTTGTACACA	CGATCCGAGGGCCTCACTA
Acaa1a	TCTCCAGGACGTGAGGCTAAA	CGCTCAGAAATTGGGCGATG
Acads	CCCAACCTGCTTGTCTCCTTG	ATCCCTGGATCACCGATTTCT
ACC-1	GATGAACCATCTCCGTTGGC	GACCCAATTATGAATCGGGAGTG
ApoB	AAGCACCTCCGAAAGTACGTG	CTCCAGCTCTACCTTACAGTTGA
ATF4	GAGTCTGAAGTCGGGACCAC	GATCGTCGGCTGGAACAC
ATF6	TCGCCTTTTAGTCCGGTTCTT	GGCTCCATAGGTCTGACTCC
BAP31	GCCACCTTCCTCTACGCAG	TGCCATAGGTCACTACCAACTC
CHOP	ACCTTCACTACTCTTGACCCTG	GATGTGCGTGTGACCTCTGT
CPT1a	CTCCGCCTGAGCCATGAAG	CACCAGTGATGATGCCATTCT
FATP2	TCCTCCAAGATGTGCGGTACT	TAGGTGAGCGTCTCGTCTCG
FATP5	CTACGCTGGCTGCATATAGATG	CCACAAAGGTCTCTGGAGGAT
FAS	GGAGGTGGTGATAGCCGGTAT	TGGGTAATCCATAGAGCCCAG
GRP78	ACTTGGGGACCACCTATTCCT	ATCGCCAATCAGACGCTCC
HMG-CoA Syn	GCCGTGAACTGGGTCGAA	GCATATATAGCAATGCTCCTGCAA
HMG-CoA Red	GATCTGGCAGTCAGTGGGAA	GTTGTAGCCGCCTATGCTCC
IRE1α	ACACTGCCTGAGACCTTGTTG	GGAGCCCGTCCTCTTGCTA
LDLR	AGTGGCCCCGAATCATTGAC	CTAACTAAACACCAGACAGAGGC
MTTP	CTCTTGGCAGTGCTTTTTCTCT	GAGCTTGTATAGCCGCTCATT
XBP1	AGCAGCAAGTGGTGGATTTG	GAGTTTTCTCCCGTAAAAGCTGA
XBP1s	GAGTCCGCAGCAGGTG	GTGTCAGAGTCCATGGGA
SCD1	TTCTTGCGATACACTCTGGTGC	CGGGATTGAATGTTCTTGTCGT
SREBP1C	GCAGCCACCATCTAGCCTG	CAGCAGTGAGTCTGCCTTGAT
SREBP2	GTGGGAGAGTTCCCTGATTG	CTCCACCATTGTTGCCTCTG
VLDLR	TGACGCAGACTGTTCAGACC	GCCGTGGATACAGCTACCAT

Table S2. List of antibodies used in this study.

Antibody	Isotype	Cat#	Source	Dilution
ACC-1	Rabbit IgG	3676	CST	1:2000
p-ACC-1	Rabbit IgG	3661	CST	1:3000
ATF4	Rabbit IgG	sc-22800	Santa Cruz	1:1000
ATF6	Rabbit IgG	sc-22799	Santa Cruz	1:1000
BAP31	Goat IgG	ab10924	Abcam	1:2000
CHOP	Mouse IgG	2895	CST	1:1000
c-Jun	Rabbit IgG	9165	CST	1:1000
eIF2α	Mouse IgG	sc-133227	Santa Cruz	1:1000
p-eIF2α	Rabbit IgG	3398	CST	1:1000
FAS	Rabbit IgG	3180	CST	1:2000
GRP78	Rabbit IgG	3183	CST	1:2000
JNK	Rabbit IgG	9252	CST	1:1000
p-JNK	Rabbit IgG	4668	CST	1:1000
Lamin B1	Mouse IgG	ab8982	Abcam	1:1000
NK-κB	Rabbit IgG	8242	CST	1:1000
PDI	Rabbit IgG	3501	CST	1:3000
SREBP1C	Mouse IgG	ab3259	Abcam	1:1000
GAPDH	Rabbit IgG	2118	CST	1:5000

CST: cell signaling technology.
